# Infection, recovery and re-infection of farmed mink with SARS-CoV-2

**DOI:** 10.1371/journal.ppat.1010068

**Published:** 2021-11-15

**Authors:** Thomas Bruun Rasmussen, Jannik Fonager, Charlotte Sværke Jørgensen, Ria Lassaunière, Anne Sofie Hammer, Michelle Lauge Quaade, Anette Boklund, Louise Lohse, Bertel Strandbygaard, Morten Rasmussen, Thomas Yssing Michaelsen, Sten Mortensen, Anders Fomsgaard, Graham J. Belsham, Anette Bøtner

**Affiliations:** 1 Department of Virus and Microbiological Special Diagnostics, Statens Serum Institut, Copenhagen S, Denmark; 2 Department of Veterinary and Animal Sciences, University of Copenhagen, Frederiksberg C, Denmark; 3 Department of Chemistry and Bioscience, Aalborg University, Aalborg, Denmark; 4 Danish Veterinary and Food Administration, Glostrup, Denmark; The Peter Doherty Institute and Melbourne University, AUSTRALIA

## Abstract

Mink, on a farm with about 15,000 animals, became infected with SARS-CoV-2. Over 75% of tested animals were positive for SARS-CoV-2 RNA in throat swabs and 100% of tested animals were seropositive. The virus responsible had a deletion of nucleotides encoding residues H69 and V70 within the spike protein gene as well as the A22920T mutation, resulting in the Y453F substitution within this protein, seen previously in mink. The infected mink recovered and after free-testing of 300 mink (a level giving 93% confidence of detecting a 1% prevalence), the animals remained seropositive. During further follow-up studies, after a period of more than 2 months without any virus detection, over 75% of tested animals again scored positive for SARS-CoV-2 RNA. Whole genome sequencing showed that the viruses circulating during this re-infection were most closely related to those identified in the first outbreak on this farm but additional sequence changes had occurred. Animals had much higher levels of anti-SARS-CoV-2 antibodies in serum samples after the second round of infection than at free-testing or during recovery from initial infection, consistent with a boosted immune response. Thus, it was concluded that following recovery from an initial infection, seropositive mink were readily re-infected by SARS-CoV-2.

## Author summary

Early on, in the course of SARS-CoV-2 infections among mink in Denmark, we identified a farm, with about 15,000 mink, that had virus-infected animals. At this time, we found that a very high proportion of the mink had been infected and made antibodies against the virus. In contrast to the three previously infected farms, the mink were allowed to recover (the mink had shown few signs of disease and only low mortality) and our testing demonstrated the absence of circulating virus. Continued screening, in the following weeks, supported the absence of infection in the mink but the maintenance of antibodies against the virus. However, less than 3 months after the initial infection, we again identified the presence of virus in some dead mink from this farm and in many live mink. The viruses responsible for this second wave of infection were slightly different from those found in the first wave but were closer to each other than to the SARS-CoV-2s found on other mink farms. The antibody levels in mink during this second wave of infection were much higher than observed after the initial infection. We concluded that the initial round of infection in mink was insufficient to confer protection against re-infection.

## Introduction

The SARS-CoV-2 has caused a pandemic with about 200 million cases reported and it has contributed to the deaths of over 5 million people [1]. Farmed mink (*Neovison vison*) are also highly susceptible to infection by SARS-CoV-2 [2, 3]. As in humans, the infection in mink can cause respiratory distress and, in some cases, mortality. However, often the proportion of infected mink that show clinical disease is low. Cases of SARS-CoV-2 infection in farmed mink were initially observed in the Netherlands (NL), in April 2020 [3], and then independently in Denmark (DK) in June 2020 (note, different clades of the virus were involved, [2]). Outbreaks continued and about 70 farms in the NL became infected [4], while 290 farms out of about 1200 mink farms in DK were found positive for the virus [5]. All farmed mink (>15,000,000) in DK have now been culled [6]. Similarly, the termination of mink farming in the NL was brought forward by 3 years from the previous plan of 1^st^ January 2024 [4]. The routes of transmission of the virus between mink farms are not fully understood [5] but it has become apparent that spread of the virus can occur not only from humans to mink but also from mink to humans [2, 7].

After the initial cases of SARS-CoV-2 infection in mink in DK, on Farms 1–3 in Northern Jutland [2], a screening program was established to test dead mink from all Danish mink farms for the presence of SARS-CoV-2, every 3^rd^ week [6]. Infection of mink on Farm 4 was identified through this Early Warning (EW) program but, in contrast to Farms 1–3, the mink on this farm were not culled and the seropositive animals apparently cleared the infection. This allowed an evaluation of the duration and efficacy of the immune response in mink to protect against re-infection.

## Results

### Infection of mink on Farm 4

Farm 4 (with about 2400 adult mink and 12600 kits in 24 open-sided houses, S1 Fig), was located near Hjørring (in Northern Jutland). It had been classified as having “Aleutian disease-free status” since 2015. This farm was tested for the presence of the SARS-CoV-2 as part of the EW screening program. On 20^th^ July 2020, 5 dead mink from this farm were tested for the virus and all were RT-qPCR negative. However, on 11^th^ August, a further 5 dead mink were tested and all were positive in this assay (Table 1). In follow-up testing, on 13^th^ August, 23 of 30 live mink tested (16 adults and 14 kits) were positive. A further 7 (of 10) dead mink also tested positive. All live mink tested (30 kits and 30 adults) were also strongly seropositive on 19^th^ August, but a reduced proportion of the mink (13 of these 60 mink tested) were positive for SARS-CoV-2 RNA. However, throat swab samples from 21 dead mink were all positive for viral RNA. Furthermore, on 31^st^ August, 7 out of 24 dead mink also tested positive by RT-qPCR. The mink on the farm were not culled but closely followed and, from 15^th^ September onwards, no virus was detected by RT-qPCR among the mink. For “free-testing”, on 30^th^ September, a larger sampling was performed involving some 300 animals, from across each of the different animal houses. All the samples were tested (in 60 pools of 5 samples) with negative results (Table 1). At least 12 samples were collected from each of the 24 houses, from adults and kits in similar proportions. This testing was designed to detect, with 95% confidence, a 1% prevalence of SARS-CoV-2 RNA positive animals. Hence, the infection had apparently disappeared among the mink on this farm at that time.

**Table 1:** Summary of laboratory analysis of mink sampling from Farm 4.

	ELISA		RT-qPCR		
Sample origin	Sera (positive/tested)	%	Throat swabs (positive/tested)	%	Date of sample collection
Dead mink (EW)	n.d.		0/5	0	20-07-2020
Dead mink (EW)	n.d.		5/5	100	11-08-2020^1^
Live adult mink	n.d.		11/16	69	13-08-2020
Live mink kits	n.d.		12/14	86	13-08-2020
Dead mink	n.d.		7/10	70	13-08-2020
Live adult mink	30/30	100	4/30	13	19-08-2020
Live mink kits	30/30	100	9/30	30	19-08-2020
Dead mink	n.d.		21/21	100	19-08-2020
Dead mink	n.d.		7/24	29	31-08-2020
Dead mink	n.d.		0/31	0	15-09-2020
Dead mink	n.d.		0/25	0	28-09-2020
Live mink	n.d.		0/60*	0	30-09-2020
Live adult mink^2^	29/29	100	0/29	0	05-10-2020
Live mink kits^2^	30/30	100	0/30	0	05-10-2020
Dead mink (EW)	n.d.		1/2**	50	02-11-2020
Dead mink (EW)	n.d.		1/2**	50	04-11-2020
Live mink	30/30	100	23/30	77	06-11-2020
Dead mink	n.d.		3/5	60	06-11-2020

n.d.: not done

*300 animals were tested in pools of 5, i.e. in 60 assays

** two pools of 5 samples tested

1: Samples were received at SSI on this date.

2: Samples were collected from the same animals as 19-08-2020

**Fig 1:**
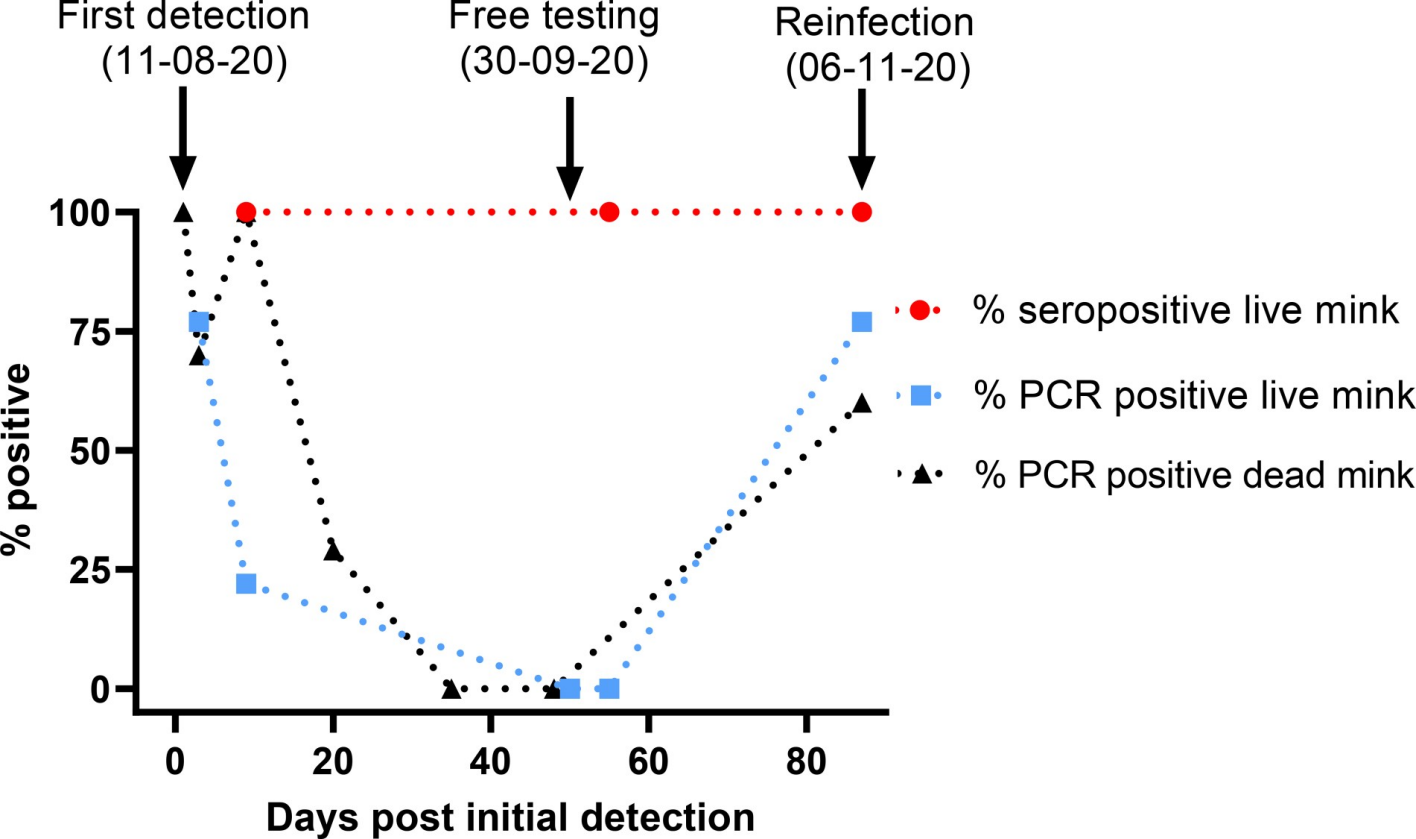
Timeline for infection of mink on Farm 4. The percentage of live and dead mink assayed by RT-qPCR that tested positive is shown throughout the period of August to November 2020, together with the proportion of live mink that tested positive by ELISA for anti-CoV-2 antibodies. The numbers of animals tested on each date are shown in Table 1. Dotted lines have been used to connect the data points for clarity but should not be used to infer intermediate percentage levels.

Surveillance of the farm continued and, in early October, 59 of 60 live mink tested in mid-August were again all found negative by RT-qPCR but all these mink remained seropositive (Table 1). Thus, no animals tested positive by RT-qPCR in September and October but all tested animals had antibodies against SARS-CoV-2. However, unexpectedly, 1 of 2 pools of 5 dead mink tested, as part of the continuing EW program, on both 2^nd^ and 4^th^ November were found to be positive by RT-qPCR (Table 1). Consequently, during the culling process that was initiated, a further 30 animals were tested on 6^th^ November and 23 (77%) were found SARS-CoV-2 RNA positive and 100% of these animals were found to be seropositive. In addition, 3 out of 5 additional dead mink were found positive by RT-qPCR (Ct values for 15 of 23 samples were below 30). During the initial infection, the farmer noticed reduced feed intake with some cases of diarrhea among the mink, and a few mink later displayed respiratory distress, however, no respiratory signs were displayed before culling during the reinfection. Fig 1 shows a timeline for the percentages of the mink tested that were seropositive and RT-qPCR positive on Farm 4 during the period August-November 2020.

### Assessment of anti-SARS-CoV-2 antibodies in mink sera by ELISA

**Fig 2:**
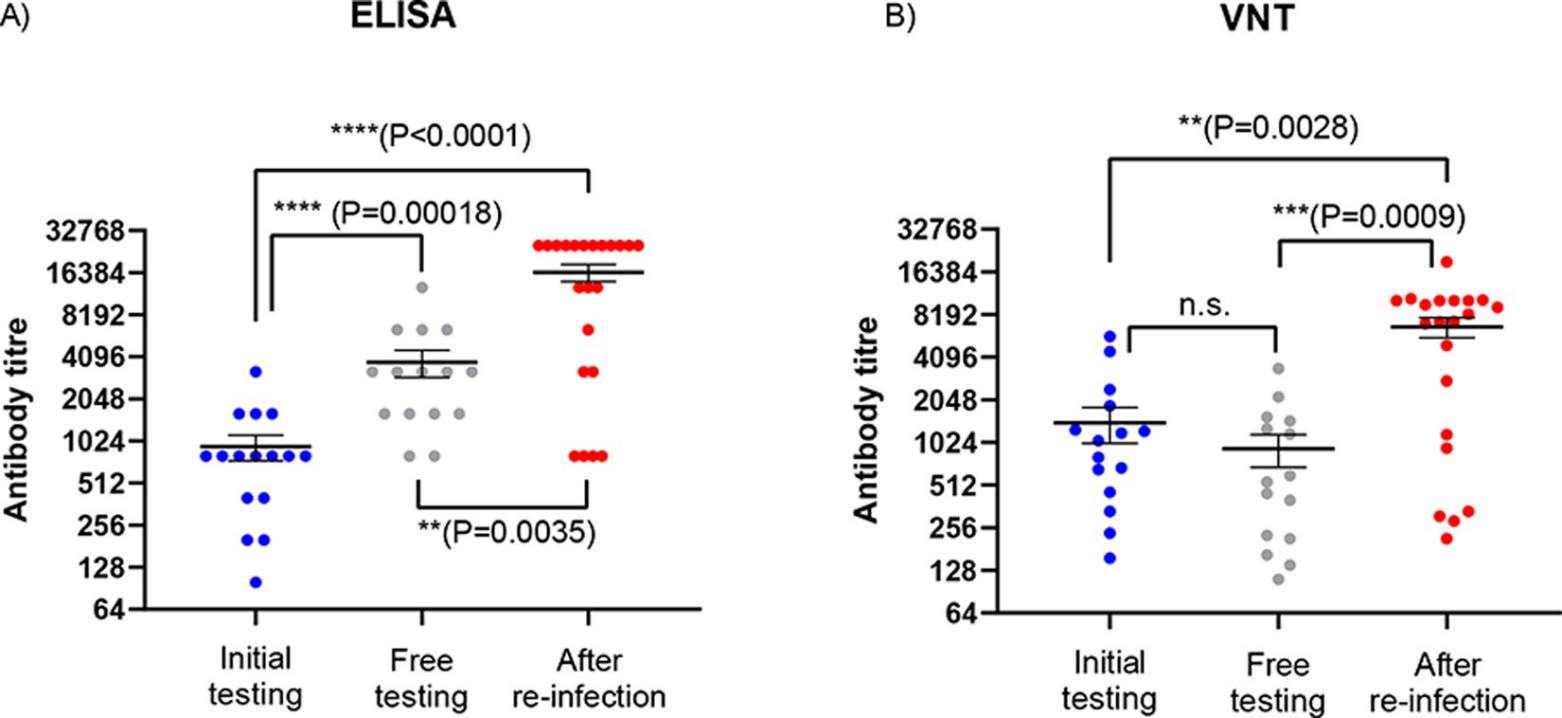
Assessment of anti-SARS CoV-2 antibody levels in mink sera. Panel A. Anti-SARS-CoV-2 antibody titres were measured by ELISA. Selected sera from mink collected at the time of initial diagnosis (blue circles), at free-testing (grey circles) and following re-infection (red circles), on 19-08-20, 05-10-20 and 06-11-20 respectively, that scored positive when assayed undiluted were titrated and assayed again by ELISA. The reciprocals of the highest dilution yielding a positive signal are plotted (on a log2 scale). Mean (+/- SEM) values are indicated by horizontal black lines. Panel B. The same serum samples were also assayed in virus neutralization assays and the calculated antibody titres are plotted (on a log2 scale) using the same colour scheme. The statistical significance of the differences between anti-CoV-2 antibody levels was determined using the Kruskal-Wallis test (see Materials and Methods).

Titration, using ELISA, of the anti-SARS-CoV-2 antibodies (that recognize the receptor binding domain (RBD)) in seropositive serum samples collected from August onwards showed that much higher levels of these antibodies were present in the mink in November, following the second round of infection than in August or October (Fig 2A). However, the proportion of seropositive animals among the tested mink had been high throughout. In August (at initial testing), the median antibody titre observed (from 16 animals tested) was 800 (range from 100 to 3200), with just a single animal having the highest titre. In early October (at free-testing), the titres in 15 sera tested were higher (ranging from 800 to 12800, with 4 of the sera having titres of ≥6400, the median titre was 3200). However, in November, following re-infection as judged by the reappearance of RT-qPCR positive animals, 16 of 22 sera tested, had titres ≥6400 and 12 had titres of 25600, see Fig 2A). There were significant differences in the levels of antibodies detected by ELISA in the serum samples collected at different times (P < 0.001, Kruskal-Wallis test), with higher levels at the time of “free-testing” compared to initial sampling and much higher levels following reinfection compared to the earlier testing.

### Assessment of neutralizing antibodies

To assess whether the anti-SARS-CoV-2 antibodies in the mink that were detected by ELISA could neutralize virus infectivity, the same serum samples were also tested in virus neutralization assays using a human SARS-CoV-2 isolate that had the same amino acid changes in the spike protein as the viruses identified initially on Farm 4 (as used previously [8]). The results (Fig 2B) showed a similar profile of antibody levels as observed in the ELISA. All of these ELISA positive sera tested had neutralization activity but the levels of these antibodies were greatly elevated after the re-infection (sera collected in November). This difference in levels of neutralizing antibodies between the time of “free testing” and after reinfection was statistically significant (P = 0.0009), while there was no significant difference in levels of neutralizing antibodies between the time of initial testing and the time of free testing (P = 0.38). There was a high degree of correspondence between the levels of antibodies detected in the two different types of assay (for all samples, the Spearman correlation co-efficient r = 0.793, P <0.0001).

### Whole genome sequencing of viruses on Farm 4

**Fig 3:**
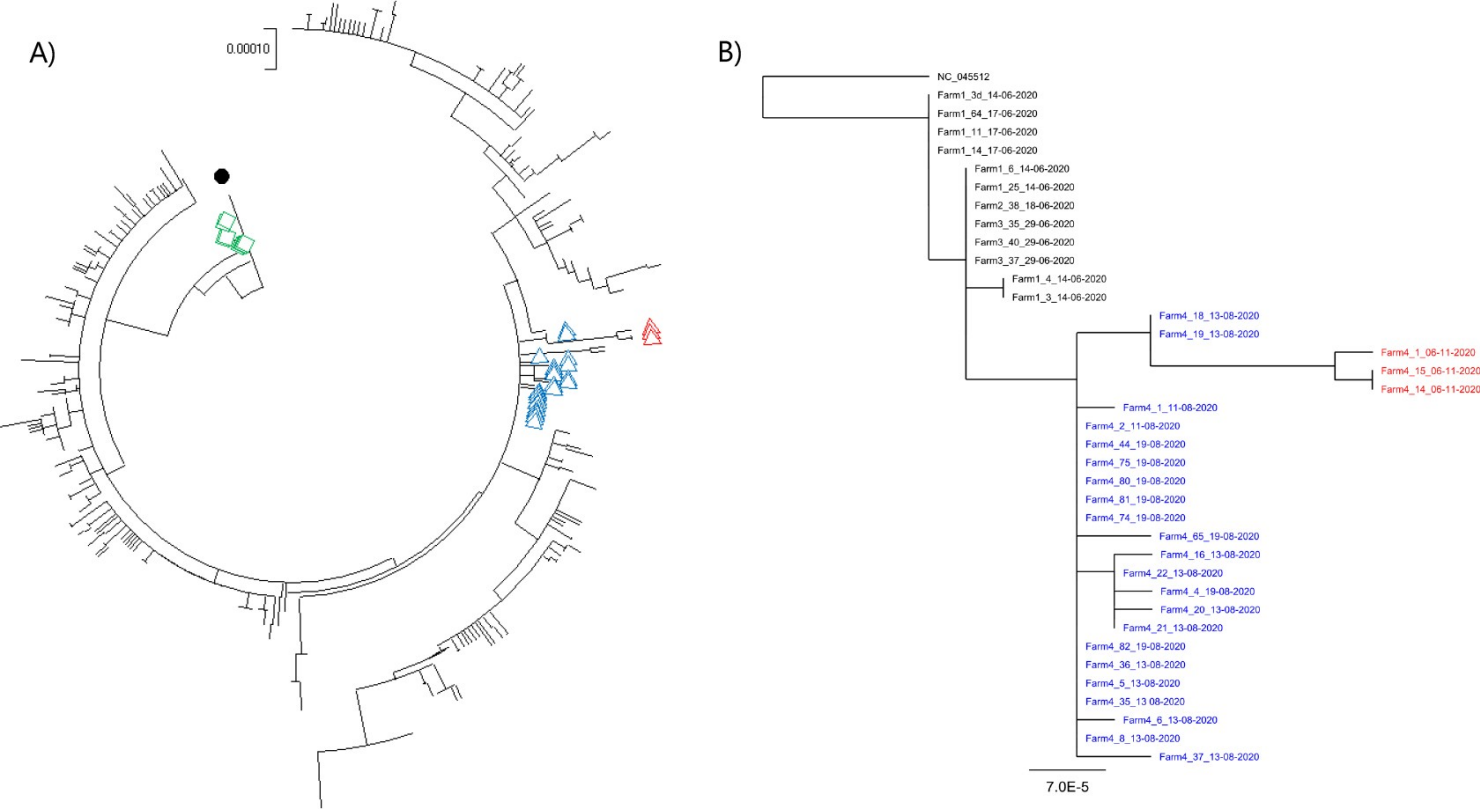
Phylogenetic trees showing the relationships between the full genome sequences of SARS-CoV-2 samples from Danish mink farms with the lineage B.1.1.298 variants. Panel A. All known SARS-CoV2 lineage B.1.1.298 genome sequences (436 in total) from Danish mink along with the Wuhan reference sequence (NC_045512.2) were included in this analysis. Sequences from the re-infection on Farm 4 (collected in November) are indicated by open red triangles while samples collected in August are indicated by blue triangles. Sequences from Farm 1 are indicated with green squares and the Wuhan reference strain with a black filled circle. The GISAID accession IDs are listed in S1 Table. Panel B. The Maximum Likelihood phylogenetic relationships between the viruses collected on Farm 4 and those from Farms 1–3 are shown. The GISAID accession IDs are listed in S2 Table.

The complete genome sequences of the viruses, from multiple samples, from infected mink in August and in November were determined. The viruses on Farm 4 in August were very closely related to the viruses that were previously identified on Farms 1, 2 and 3 [2], they were from clade 20B / lineage B.1.1.298 (Fig 3A and 3B and Table 2) and appeared part of the same transmission chain. In particular, they each had the mutation A22920T in the spike protein coding sequence, resulting in the amino acid substitution Y453F. However, in addition to this change, the spike protein gene in the viruses on Farm 4 had a deletion of 6 nt (Δ21766–21771). This affected 3 codons, changing GCT.ATA.CAT.GTC.TCT to GCT.ATC.TCT, the encoded amino acid sequence is changed from A-I-H^69^-V^70^-S to A-I-S thus residues H69 and V70 in the N-terminal domain (NTD) of the spike protein are lost. This deletion had not been identified previously in mink or in humans in combination with the Y453F substitution (see Table 2) but the deletion of these residues is shared with the SARS CoV-2 variant of concern (VOC) 202012/01 [9]. Two other deletions in the ORF1a coding sequence (Δ517–519 and Δ6510–6512) and two other amino acid substitutions (P3395S in ORF1a and S2430I in ORF1b) were also observed in some of the viruses present in the mink during this initial infection in August. The viruses present on Farm 4 in November were most closely related to those seen previously on Farm 4, over 2 months earlier (Fig 3A and 3B). It should be noted that, by November 2020, over 200 farms in DK had been identified as having infected mink [5] and a number of different variants had been observed in the animals [6]. The viruses on Farms 1–3 were closely related to each other and also to the viruses present in August on Farm 4, but the latter viruses had some additional changes (e.g. the deletion of residues H69 and V70 in the spike protein, see Table 2), which were also found in most of the later outbreaks in farmed mink in DK. Thus, viruses in farms infected after Farm 4 (identified on August 11^th^) were nearly all derived from those first detected on Farm 4. As indicated above, the November viruses (following re-infection) from Farm 4 had the A22920T mutation and the deletions in the S and ORF1a coding sequences. However, they also had additional changes across the genome, both within and outside of the S gene, compared to the viruses from Farm 4 in August (Table 2). It is noteworthy that the Farm 4 sequences in November had changes at nt 10448 (encoding the substitution P3395S in ORF1a) and 20756 (encoding S2430I in ORF1b) that had only been seen in a subset of the August sequences from Farm 4 (samples Farm4_18_13-08-2020 and Farm4_19_13-08-2020, see Fig 3B). These changes strongly suggest that the infection in November was not due to an entirely new introduction of virus into the farm from elsewhere (Fig 3A). Furthermore, the viruses on Farm 4 in November also all shared changes at nt 3792 (resulting in A1176V), 5167, 10887 (resulting in G3541E), 21727 and 23815 (these latter two silent changes are in the S gene) that were not present in any of the Farm 4 sequences in August (Table 2). The presence of these additional sequence changes indicates that the virus had been replicating between August and November.

**Table 2:** Sequence changes within SARS-CoV-2 in mink on Farm 4.

Location	5’-UTR	ORF1a				ORF1b			S			ORF3a	N	
Nt	241	3037	5144	10448	11776	14408	15656	20756	Δ21766–21771	22920	23403	25936	28854	other
Virus														
Wuhan	C	C	C	C	C	C	C	G	-	A	A	C	C	
EPI_ISL_455326 20B	T	T	C	C	C	T	C	G	-	A	G	C	C	
Farm 1	T	T	C	C	C	T	T	G	-	T/A	G	T	C	
Farm 2	T	T	C	C	C	T	T	G	-	T	G	T	C	
Farm 3	T	T	C	C	C	T	T	G	-	T	G	T	C	
**Aug 2020**														
Farm4_5	T	T	T	C	T	T	T	G	+	T	G	T	T	
Farm4_6	T	T	T	C	T	T	T	G	+	T	G	T	T	G488A
Farm4_8	T	T	T	C	T	T	T	G	+	T	G	T	T	Δ21984–21995
Farm4_18	T	T	T	T	T	T	T	T	+	T	G	T	T	
Farm4_19	T	T	T	T	T	T	T	T	+	T	G	T	T	
Farm4_21	T	T	T	C	T	T	T	G	+	T	G	T	T	A652C (K129N)^1^
Farm4_35	T	T	T	C	T	T	T	G	+	T	G	T	T	Δ27982–28030
Farm4_37	T	T	T	C	T	T	T	G	+	T	G	T	T	T1873C,G2035T(L590F)
**Nov 2020**														
Farm4_1	T	T	T	T	T	T	T	T	+	T	G	T	T	C1913T (R550C), C3792T(A1176V), C5167T, G10887A(G3541E), C21727T, T23815C
Farm4_14	T	T	T	T	T	T	T	T	+	T	G	T	T	A3303G, C3792T(A1176V), C5167T, G10887A(G3541E), C21727T, T23815C
Farm4_15	T	T	T	T	T	T	T	T	+	T	G	T	T	A3303G, C3792T (A1176V), C5167T, G10887A(G3541E), C21727T, T23815C
AA change	-	-	-	P3395S	-	P314L	T730I	S2430I	ΔH69-V70	Y453F	D614G	H182Y	S194L	

1: Note the same additional sequence change was also present in 4 other samples (Farm4_16_13-08-2020, Farm4_20_13-08-2020, Farm4_22_13-08-2020 and Farm4_4_19-08-2020). N.B. All the mink viruses, together with the EPI_ISL455326 clade 20B representative sequence, shown here were from clade 20B and had the changes G28881A, G28882A and G28883C compared to the Wuhan strain. In addition, the mink viruses from Farm 4 also lacked nt 517–519 and nt 6510–6512. Nucleotide changes from the Wuhan reference sequence are highlighted in yellow. Shared additional changes that occurred in viruses on Farm 4 between August and November 2020 are indicated with colour codes, encoded amino acid changes, where applicable, are also shown. The Accession IDs for all the indicated sequences are listed in S2 Table.

Phylogenetic analysis clearly showed that all viruses from Farm 4 were very closely related to each other, including the viruses from both August and November (Fig 3B). As described above, two of the early Farm 4 viruses (Farm4_18_13-08-2020 and Farm4_19_13-08-2020) shared additional changes at nt 10448 and 20756 (see Table 2) and the November viruses formed their own distinct branch from these (Fig 3B), due to the presence of the further sequence changes (Table 2).

## Discussion

SARS-CoV-2 can readily infect humans and mink. In addition, certain other species, e.g. cats, dogs and ferrets, can also be infected following direct inoculation under experimental conditions [10, 11]. Furthermore, some cases of transmission from infected people to their cats and dogs have occurred but it does not seem to happen more generally. Both cellular and humoral immune responses occur within SARS-CoV-2-infected people and animals [12, 13] and it is common for both humans and animals to be both seropositive and RT-qPCR positive simultaneously (see [2, 14]). However, as people and animals recover, the levels of virus subside but antibody levels persist, or increase, at least for some time.

Farm 4 was the first Danish mink farm, where the animals were allowed to recover following SARS-CoV-2 infection and were then tested with the purpose of documenting freedom from the virus about 2 months after the initial infection. Thus, Farm 4 gave a unique opportunity to follow the maintenance of anti-SARS-CoV-2 antibodies over an extended period and the resistance of the animals to reinfection. As also observed on other mink farms in DK [5], very widespread infection of the mink on Farm 4 by SARS-CoV-2 occurred in the first wave of infection, with 100% of the tested animals being seropositive. During the following period of over 2 months, the animals were repeatedly screened and shown to be negative by RT-qPCR, while 100% of the tested mink remained seropositive. However, in November, it was found that the mink had become infected again. Surprisingly, a high proportion (>75%) of the animals tested had been re-infected by SARS-CoV-2 (Table 1 and Fig 1). The virus responsible for the second round of infection was most closely related to the virus found almost 3 months earlier on this farm (Fig 3A and 3B), but with distinctive differences (see Table 2) from the viruses responsible for the initial infections observed in mink on Farms 1–3 [2]. The virus acquired sequence changes during the period between the infections recognized in August and November (Table 2), indicative of continued replication, rather than simply having been preserved in an infectious form. Since the viruses present on Farm 4 in November were most closely related to viruses present on the same farm in August, it seems most likely that re-infection of the mink from within the farm had occurred. It cannot be established, however, whether the virus had continued to replicate in a small number of mink on the farm, but with very restricted spread, or if it had replicated in an alternative host, linked to the farm, during this time and had then been re-introduced into the seropositive mink. It has been demonstrated, in both DK and the NL, that transmission between humans and mink can occur in both directions [2, 6]. Transmission to, and from, other host species is theoretically possible (but not described previously; some cats were found to be infected on mink farms in DK and in the NL but they do not seem to spread the virus). It has been found that there was a cluster of occurrences of SARS-CoV-2 with the ΔH69/N70 and Y453F changes (as in Farm 4) in the local human population in August. Furthermore, a virus containing these changes plus the additional mutations (i.e. C3792T, C5167T, G10887A, C21727T and T23815C, see Table 2), which were present in the mink viruses from Farm 4 in November, was found in one person in early November. It seems likely that these human cases were infections derived from mink.

A high proportion of the sequence changes observed in mink (Table 2), which occurred in the viruses from Farm 4 between August and November (and also between the clade 20B viruses and the Wuhan virus, see [2]), involved C to T changes (in cDNA) that correspond to C to U changes in the viral RNA. Several of these nt changes are synonymous, i.e., they do not result in amino acid sequence changes. It has been suggested that such changes reflect host immune pressure via RNA editing systems (e.g. by APOBEC) rather than selection for increased transmissibility in particular hosts [15–17]. However, this process of RNA editing is not relevant to the key mutation in the S gene (A22920T), which seems to be an adaptation that occurred during the initial infection of mink [2], or to the generation of deletions. The loss of residues H69 and V70 in the spike protein, seen in mink for the first time on Farm 4, and in certain variants from people, has been reported to double the infectivity of pseudoviruses displaying the mutant spike protein compared to the wild type particles [18]. This deletion appears to emerge in SARS-CoV-2 following other changes in the spike protein [19].

The sampling of the mink on Farm 4 tested, at most, 300 animals on any particular date, out of a population of about 15,000 animals. It is clearly possible that a small number of infected mink were missed although the repeated follow-up screening makes this unlikely. The level of seropositivity among tested animals, prior to the second round of infection, remained very high (100%). Thus, it is not clear why so many animals (77% of 30 animals tested) were susceptible to a second round of infection. It has been considered whether the seropositivity detected in kits in August may be a consequence of maternally derived antibodies that could potentially decline more rapidly than antibodies generated from the infection in each animal. However, it seems difficult to reconcile this with the fact that >80% of throat swabs from mink kits tested clearly positive by RT-qPCR in August, which indicated a high level of infection amongst the kits in the first wave also.

The measurements of antibody responses were made using an ELISA that targets the receptor binding domain (RBD) of the spike protein. Antibodies to the SARS-CoV-2 spike protein were present in up to 100% of the infected mink. The antibody titres, measured in this assay, increased to very high levels during the period of re-infection (Fig 2A). In studies on human sera, samples testing clearly positive (10 x cut-off) in this ELISA all had neutralizing antibodies [20]. Indeed, assessment of the same samples of mink sera as tested by ELISA in virus neutralization tests indicated a high correspondence between these two types of assay. Thus, the ELISA positive mink sera neutralized the virus and, furthermore, the sera collected in November, after reinfection, had significantly higher levels of anti-SARS-CoV-2 antibodies as measured in each assay (Fig 2A and 2B), these results are consistent with boosting of the immune response due to a second round of infection.

Due to the relatively low number of samples tested, the calculated proportions of virus or antibody positive animals may not directly correspond to prevalence of infection on the farm. However, during the course of the SARS-CoV-2 infections in mink in Denmark, the collection of samples from 62 Danish mink farms, where no clinical signs were present, a prevalence of virus of 100% was found on 45% of the farms, and on only 11 farms (18%) was a prevalence of virus below 50% observed [5]. On 40 farms, where blood samples were collected at the first sampling date, i.e. shortly after suspicion of infection was raised, a seroprevalence of 100% was found in 22 farms (55%), while on 12 farms (30%), a seroprevalence below 50% was observed [5]. This indicates that when animals were entirely randomly selected (due to the complete absence of clinical signs) for sampling, high prevalence of virus as well as of seropositive animals was observed.

In Denmark, mink were usually kept in long rows of open houses, i.e. in contiguous wire netting cages (S1 Fig). Cages were typically side-by-side, with the possibility of neighbouring mink having nose-to-nose contact and of air exchange between cages. Samples of air, fur and straw from mink farms have previously been found PCR-positive for SARS-CoV-2 [3, 5, 21], indicating that transmission indirectly through feed, bedding and airborne dust might occur within farms.

It appears that the virus responsible for the infections in November was antigenically identical to the virus in August since there were no non-synonymous changes within the spike protein gene during this time, although some silent sequence changes (i.e. C21727T and T23815C) had occurred in it, as well as changes elsewhere within the virus genome.

The most plausible conclusion is that infection of farmed mink with SARS-CoV-2 does not induce long-term protection against the virus. This should be compared with the situation in rhesus macaques where primary infection did protect against reinfection at about 1 month post-initial infection [13, 22] and in humans where protection from reinfection may last at least eight months [12, 23]. However, some cases of re-infection have been reported in health care workers in Brazil [24], although this occurred in people who only developed a weak immune response during the initial infection. Furthermore, only about 50% of people aged over 65 years in DK, who had been infected with SARS-CoV-2, were found to be protected against re-infection [25]. On a mink farm, a large number of animals live in close proximity to each other and, potentially, once the infection occurs in some animals then there can be a rapid increase in virus production and a strong challenge to neighbouring animals. Perhaps this is sufficient to overcome the immune response. It is notable that greatly enhanced levels of anti-SARS-CoV-2 antibodies were detected in the mink following the second round of infection (Fig 2A and 2B), but this was also observed following challenge of previously infected rhesus macaques which did not become re-infected [13, 22]. Interestingly, two doses of a spike protein-based subunit vaccine did confer protection in mink against virus challenge at 5 weeks following the second vaccination [26] but it is not known how long this resistance to infection was maintained. Similarly, in ferrets, two doses of the adenovirus-vectored S gene vaccine provided good protection against the virus [27]; however, it is noteworthy that virus challenge after a single dose of this vaccine resulted in an increase in neutralizing antibody titres whereas no such increase was observed following challenge after two doses of the vaccine. In our study, the mink did exhibit a boost in antibody levels following the re-infection, suggesting the immune response to the initial infection was inadequate to block virus replication. Currently, there are no “correlates of protection” that can be used to evaluate the immune responses in mink.

## Materials and methods

### Ethics statement

No experimental procedures were performed on animals in this study. Only routine diagnostic samples (blood samples and throat swabs) were collected from the live, farmed, animals with no harm to them. Therefore, no prior ethical approval was required.

### Sampling strategy

At the time of first detection of infection on Farm 4, the practice was that suspected farms were visited by an official veterinarian, who took throat swabs from animals with clinical signs of SARS-CoV-2, if present, or alternatively from randomly selected animals. The numbers of samples taken varied during the epidemic. Here we describe only the samples taken in Farm 4. At first suspicion, following positive detection in the EW samples, 30 samples were taken from live animals, this approach provided 95%-confidence of detecting an infection prevalence of 0.1 with a test sensitivity of 99% and a specificity of 100%. When the samples were found positive, staff from University of Copenhagen (UCPH) collected further throat swab samples as well as blood samples for serology. UCPH staff collected samples randomly from each house (S1 Fig) and randomly from the periphery and centres of the rows [5]. UCPH collected 60 samples from live animals, providing 95%-confidence of detecting a disease prevalence of 0.05 with a test sensitivity of 99% and a specificity of 100%. As increased mortality was one of the signs observed most often on infected farms [3, 5], throat swab samples from 5–21 recently deceased animals (within the previous 14 days) were collected when indicated (Table 1).

### Laboratory analyses

Blood and throat-swab samples were collected from mink (adults and kits) as indicated in Table 1. The presence of SARS-CoV-2 RNA was determined by RT-qPCR as described previously [2]. The SARS-CoV-2 Ab ELISA (Beijing Wantai Biological Pharmacy Enterprise, Beijing, China) was used as described previously [2], according to the manufacturer’s protocol (the assay has sensitivity of 97% and specificity of 100%). Undiluted sera were used to identify seropositive animals and selected sera were then diluted (2-fold steps) and re-assayed in the ELISA. Antibody titres are presented as the reciprocal of the highest dilution of the serum giving a positive result. Neutralizing antibody titres were determined as described previously [8].

For documentation of freedom from circulating virus (free-testing), 300 randomly selected mink were tested (in 60 pools of 5 samples) by RT-qPCR. This testing strategy was designed to detect, with 95% confidence, a 1% prevalence of SARS-CoV-2 RNA positive animals in 300 individual samples, leading to 93% confidence when pooling of 5 samples was taken into account.

Selected SARS-CoV-2 positive RNA samples were sequenced as described [2] and SARS-CoV-2 sequences were aligned using MAFFT [28]. Phylogenetic analysis was performed using MEGA version X, using the Maximum Likelihood method and General Time Reversible model with elimination of all sites with less than 95% coverage [29]. The GISAID accession IDs are listed in S1 and S2 Tables.

The statistical significance of the differences between anti-CoV-2 antibody levels was determined using the Kruskal-Wallis test in R [30].

## Supporting information

S1 Fig Layout of mink houses on Farm 4. The locations from which samples were collected are indicated in dark blue (for August and October sampling) or red (November sampling). The open houses (in light blue unless sampled) in both the old and new parts of the farm are numbered and the number of mink sampled from each house are shown (in parenthesis).
(TIF)

S1 Table List of accession numbers for SARS-CoV-2 sequences obtained from Danish mink farms. The names of virus samples, as used in the phylogenetic analysis (Fig 3A), together with their date of collection plus their GISAID accession ID numbers are shown.
(XLSX)

S2 Table List of accession numbers for SARS-CoV-2 sequences obtained from mink on Farms 1–4. The names of virus samples, as used in the phylogenetic analysis (Fig 3B), together with their site and date of collection plus their GISAID accession ID numbers are shown.
(XLSX)
