# Supplementary figures and images for: Infection, recovery and re-infection of farmed mink with SARS-CoV-2
Source: PLoS Pathog. 2021 Nov 15;17(11):e1010068. doi: 10.1371/journal.ppat.1010068 (PMC8629378; doi:10.1371/journal.ppat.1010068)

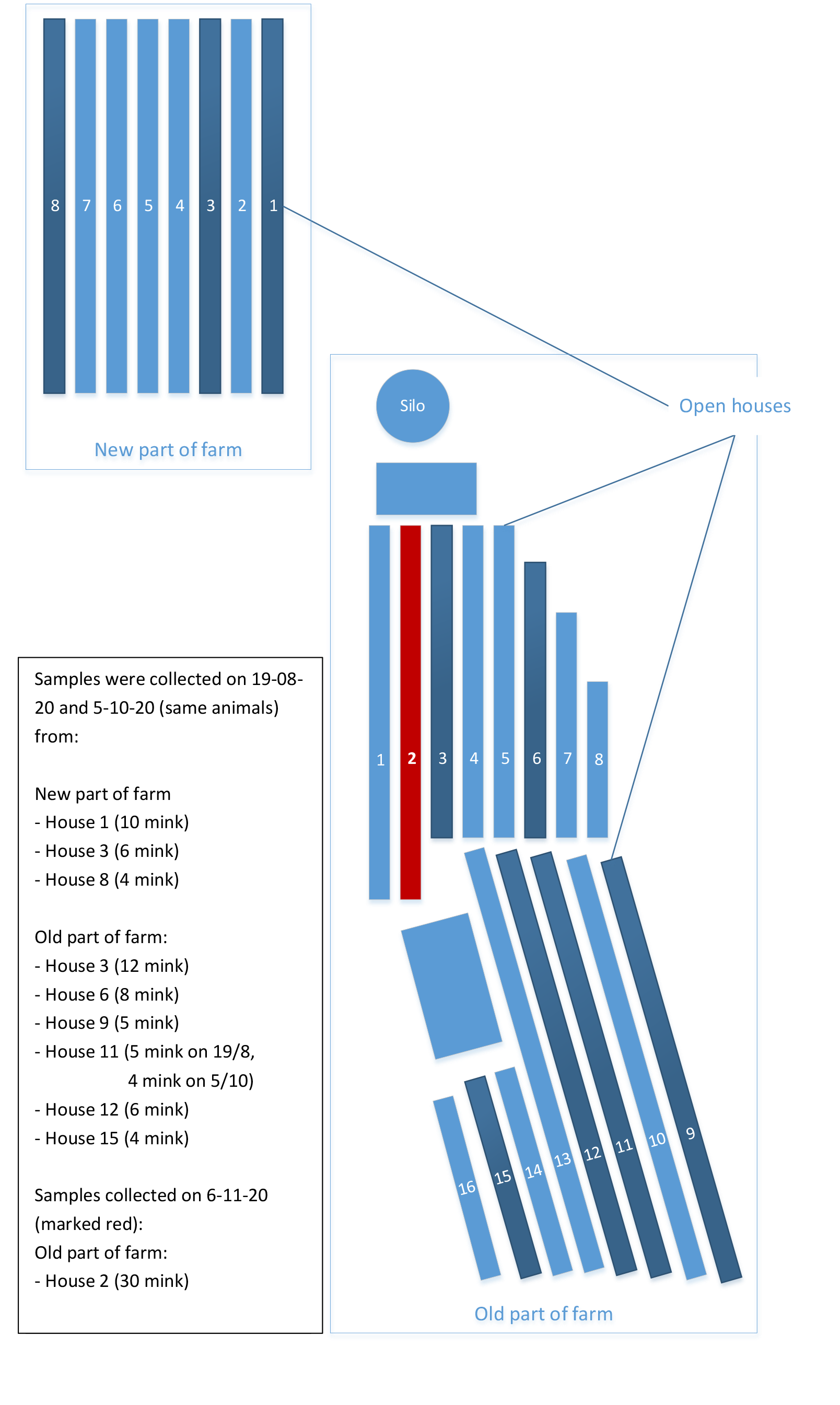

Supplement: S1 Fig — The locations from which samples were collected are indicated in dark blue (for August and October sampling) or red (November sampling). The open houses (in light blue unless sampled) in both the old and new parts of the farm are numbered and the number of mink sampled from each house are shown (in parenthesis). (TIF) [file ppat.1010068.s001.tif]
